# Quantitative Assessment of PALB2 and BRIP1 Genes Expression in the Breast Cancer Cell Line under the Influence of Tamoxifen

**DOI:** 10.31661/gmj.v12i.2483

**Published:** 2023-12-26

**Authors:** Hamed Kharrati-Koopaee, Seyed Taghi Heydari, Mehdi Dianatpour, Kamran Bagheri Lankarani

**Affiliations:** ^1^ Institute of Biotechnology, Shiraz University, Shiraz, Iran; ^2^ Health Policy Research Center, Institute of Heath, Shiraz University of Medical Sciences, Shiraz, Iran; ^3^ Department of Medical Genetics, School of Medicine, Shiraz University of Medical Sciences, Shiraz, Iran

**Keywords:** Biomarkers, Gene expression, Breast cancer

## Abstract

Background: Breast cancer is considered one of the leading causes of mortality in the world. Cancer incidence and consequently, drug consumption can strongly influence gene expressions at the transcriptome level. Therefore, the assessment of the candidate biomarkers’ gene expression can accelerate the diagnosis process and increase the chance of treatment and remission. In this regard, the quantitative assessment of Partner and localizer of BRCA2 (PALB2) and BRCA1 Interacting Helicase 1 (BRIP1) genes expression in the breast cancer cell line under the treatment of Tamoxifen (TAM) was executed in this study. Materials and Methods: MCF7 cells were cultured as TAM-treated and control groups. RNA extraction and cDNA synthesis were performed based on the instructions of provided kits. qPCR Hi-ROX Master Mix kit was applied to the quantitative real-time polymerase chain reaction (Q-PCR). The outputs of Q-PCR were analyzed by REST statistical software. Results: Outcomes derived from data analysis of BRIP1 gene expression did not show any significant difference between the gene expression of control and TAM-treated groups. The expression of PALB2 was significantly higher in the TAM-treated group compared to the control group (P0.05). Conclusion: Our findings showed a significant alteration between PALB2 gene expression in the TAM-treated breast cancer cell line and the control cell line. The quantitative assessment of mentioned genes as possible markers could be considered a non-invasive method for breast cancer in the processes of prognostic evaluations, screening, and treatment monitoring.

## Introduction

Breast cancer is known as the second leading cause of cancer-related death among females [1].

The WHO reported that in 2020, there were 2.3 million women diagnosed with breast cancer and 685,000 deaths around the world. Breast cancer is highly heterogeneous, which occurs due to the cross impacts of hereditary and environmental risk factors. In addition, it is pathologically classified based on the key protein expressions identified through immunohistochemistry [2].

Estrogen receptor (ER, ESR1 gene) that is called ER-positive (ER+), progesterone receptor (PR, PGR gene), and human epidermal growth factor receptor 2 (HER2, ERBB2 gene) are included in the aforementioned classification.

Tumors without these protein markers are called triple-negative breast cancer (TNBC) [3].

Patients with breast cancer can be treated through surgical methods, ray methods, and endocrine therapy (or anti-estrogen therapy).

Tamoxifen (TAM), the selective ER modulator (SERM) that competes with estrogens to connect to ER, as well as aromatase inhibitors (AIs), such as letrozole, are implemented in endocrine therapy to block the conversion of androgens into estrogens [4][5].

TAM is an ER antagonist, which has helped millions of females with breast cancer since 50 years ago [5].

However, it comes with several issues including drug resistance and consequent side effects. Unfortunately, 30 to 40% of patients are obstinate and show increased metastatic cancer [6][7].

Because breast cancer is a heterogeneous disease, identifying molecular markers, gene expression profiles, and genomic alteration patterns are considered analytical tools necessary for determining treatment outcomes and choosing the best treatment approaches [8][9]. Therefore, it is very important to achieve a good understanding of the cellular and molecular pathways related to breast cancer development and progression to improve the treatment conditions and clinical outputs [10].

By interacting with the BRCA2 protein, the tumor suppressor partner and localizer of BRCA2 (PALB2) plays a critical part in repairing DNA damage and preventing the growth of tumors. The mutation in the structure of PALB2 protein increases the risk of cancer by 14% and 35% among 50- and 70- year old individuals, respectively [11][12].

Similarly, the mutant protein of BRCA2 among 80-year-old females increases the risk of cancer exposure by 72%. Furthermore, BRCA1 Interacting Helicase 1 (BRIP1) encodes a protein belonging to RecQ Helicase DEAH.

Similar to PALB2, this gene is on chromosome 17 and repairs DNA damage in a complementary gene action with BRCA2. Compared to BRCA2, the importance of mutation in BRIP1 does not increase the risk of cancer exposure; however, its product is recognized as a tumor suppressor and also an oncogene protein [12][13].

In this study, the performance and alterations in gene expression of PALB2 and BRIP1, as well as their roles in cancer progression wasinvestigated through conducting quantitative assessments.

## Materials and Methods

Cancer Cells Culture and Tamoxifen Treatment Implementation

Experimental research was executed to analyze gene expression in breast cancer cell lines. MCF7 cells (National Cell Bank, Pasteur Institute of Iran) were cultured in RPMI 1640 (Bioidea Company, Iran) medium with pH 7.4.

The culture medium was supplemented with 10% heat-inactivated fetal bovine serum (Gibco-BRL, USA), 1% penicillin, and streptomycin (Biosera). Furthermore, the mentioned cells were maintained in the 5% humidified atmosphere at 37 .

Cell viability assays were also carried out by conducting the colorimetric MTT assay (Sigma-Aldrich-UK) for quantifying cell viability [14].

After 24 hours of the cell culture process, TAM (Iran-Hormone Company-Tehran, Iran) was added to the culture mediums at the density of.

The sampling procedure was conducted after 12 hours for RNA extraction.

RNA Extraction and Qualitative and Quantitative Assessments

Following the guidelines given by Denazist Asia Company, RNA was extracted from a cell culture medium. Then, the quantity and quality of the extracted RNA were assessed by 1% agarose gel electrophoresis and NanoDrop. The absorption rate of extracted RNA was assessed on the wavelengths of 280, 230, and 260, as well as rates of 260/280 and 260/230 [15].

Designing a Primer for Q-PCR

In this study, primers were designed by the gene sequence of PALB2 and BRIP1 with the accession numbers XM_017023673 and XM-011525341 available in the NCBI database. Also, Primer Quest software was implemented [14].

To assure the specificity of primers, the BLAST tool was applied in the NCBI database. Finally, the synthesis of primers was carried out by Macrogen Company, South Korea. It is noteworthy that GADPH was applied as the internal control. The primer sequences are provided in Table-1.

cDNA Synthesis and Determination of

Primers Annealing Temperature

DNaseI enzyme was used (Fermentas Company) to remove the DNA contamination from RNA extracted samples.

DNaseI treatment was carried out on the samples in 30 minutes at 37 . Finally, of 50 milli-molar EDTA was added to the solution and then, maintained at 65 for 10 minutes to inactivate the DNaseI enzyme. cDNA synthesis was conducted at the final volume of according to the kit instructions provided by Thermo Company. PCR reaction was applied to the amplification of 112 bp fragment of the GADPH gene to confirm the synthesis of

cDNAs.

The polymerase chain reaction was conducted at the volume of; also, the reaction components were autoclaved distilled water, master mix, each primer at the concentration of 0.6 picomoles, and cDNA at the concentration of 100 nanograms. The amplified product was confirmed using 1% agarose gel

electrophoresis.

Quantitative Real Time PCR

QPCR Hi-ROX Master Mix kit provided by Biocompare Company (www.biocompare.com) was applied to the Q-PCR in this study. The kit manufacturer’s instructions were considered to determine the volume of reactants. Table-2 represents the Q- PCR thermal cycles of investigated genes.

Q-PCR Data Analysis

REST software and method were used to analyze the expression data from Q-PCR [16]. The Kolmogorov-Smirnov and the Shapiro-Wilk tests were used to analyzing the normality of the expression data.

Differences between treatments with three replications including the TAM-treated cell line and TAM-free cell line were tested using the independent T-test via SPSS software (Version 16; SPSS Inc., Chicago, USA) at the significant level of 0.05 (P<0.5).

Ethics approval

This study was approved by the ethics committee of Shiraz University of Medical

Sciences (IR.SUMS.REC.1400.293).

## Results

**Table:** Table1. Sequences of Investigated Genes Primers for Gene Expression Assessment

Genes	Length of pieces (bp)	Primers sequence (5´ to 3´)
PALB2	133	F: AGGATCTCTCACCGCAGCTAA
		R:TCAGGCCCAACATCAAGTGTG
BRIP1	144	F: CTTACCCGTCACAGCTTGCTA
		R: CACTAAGAGATTGTTGCCATGCT
GADPH	112	F: CTCTCTGCTCCTCCTGTTCG
		R: ACGACCAAATCCGTTGACTC

**Table:** Table2. The Quantitative Real Time Polymerase Chain Reaction (Q-PCR) Thermal Cycles of Investigated Genes.

**Genes**	**Number of cycles **	**Time**	**Temperature (** **°** **C)**	**Stage**
***PALB2***	1	12 min	94	Initial denaturation
		1 min	94	Annealing
	40	50 sec	55	
		40 sec	72	
	1	90 sec	72	Final extension
***BRIP1***	1	3 min	95	Initial denaturation
		50 sec	95	
	40	40 sec	57	Annealing
		5 min	72	
	1	90 sec	72	Final extension

Qualitative and Quantitative Assessments of RNA extraction

**Figure-1:**
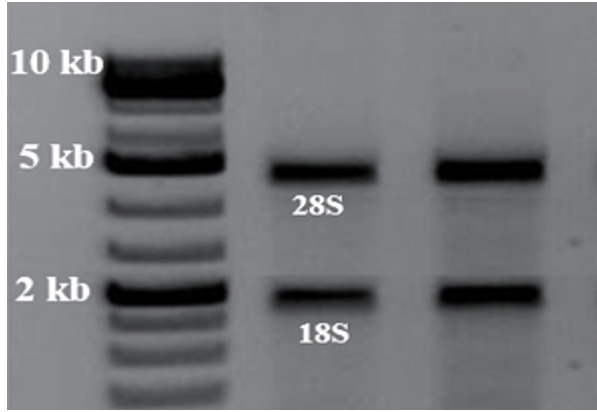
Figure-1. The confirmation of RNA extraction by gel electrophoresis.

Agarose gel electrophoresis was used to qualify RNA after extraction. The presence of 18s and 28s fragments and the absence of extra bands in the extracted samples indicated the appropriate RNA quality (Figure-1).

The quantitative assessment of extracted RNA, as well as the determination of appropriate quantities required for generating cDNA, were carried out using NanoDrop. The outcomes revealed that all RNA extracted samples have appropriate quality and quantity for cDNA synthesis.

Confirmation of cDNA synthesized

**Figure-2:**
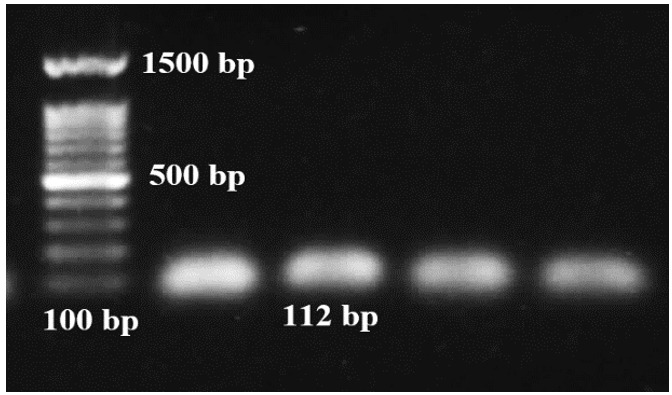
Figure-2. Electrophoresis results of amplification of GADPH gene (112 bp) in order to confirmation of cDNA synthesis.

Following Nano dropping and determining the concentrations of the samples, related cDNA was generated and then the PCR process was conducted using the internal control gene primers. Finally, the product was investigated using the agarose gel (Figure-2).

The amplification of the 112 bp fragment of the GADPH gene demonstrated that cDNA synthesis was performed correctly.

Results of Gene Expression Measurement

**Figure-3:**
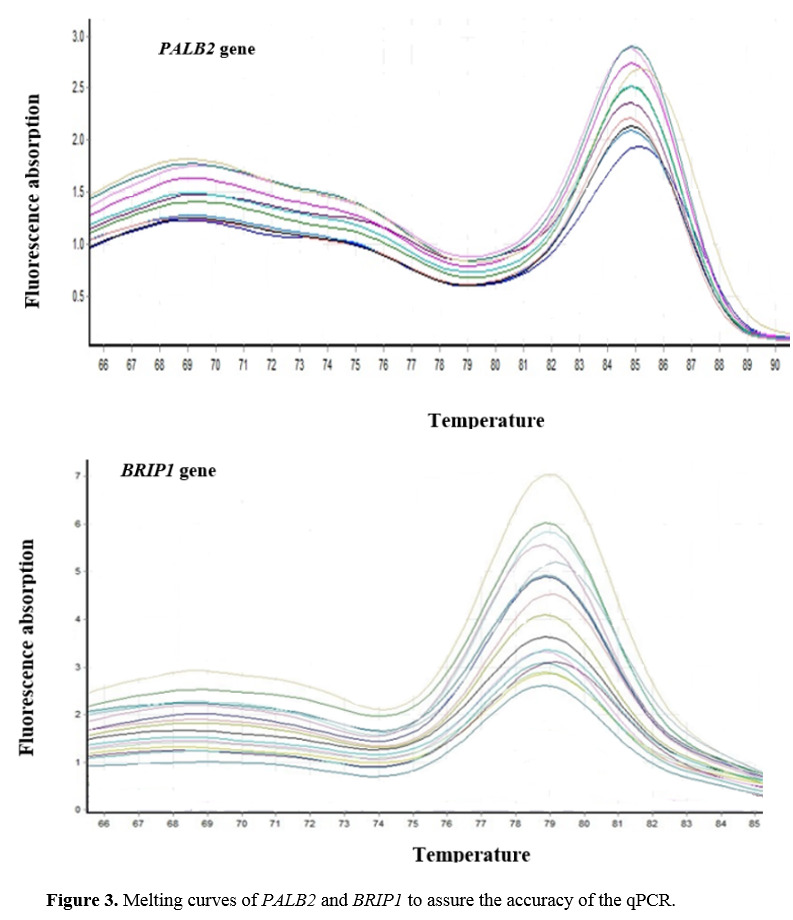
Figure-3. Melting-curves of PALB2 and BRIP1 genes to assure the accuracy of the Quantitative Real Time Polymerase Chain Reaction (Q-PCR).

The results of the melting curve were applied to ensure the Q-PCR accuracy and precise amplification of genes under investigation. A peak in the melting curve of each designed primer showed the specific amplification of genes. Figure-3 shows the melting curve of PALB2 and BRIP1 genes. The normality of expression data was approved by normality tests. Results of investigating gene expression data revealed that BRIP1 gene expression of the control and TAM-treated cell lines were not significantly different.

**Figure-4:**
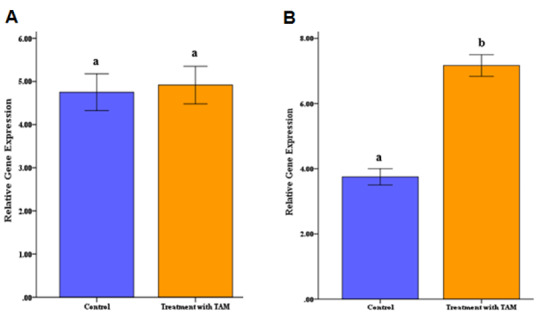
Figure-4. Differences in PALB2 and BRIP1 genes expression in Tamoxifen (TAM)-treated and control MCF7 cell line. Different letters indicate statistically significant differences at P<0.05. Bars with common letters show no significant differences.

In contrast, alterations in PALB2 gene expression of the TAM-treated and control groups were associated with a significant increase (P<0.05, Figure-4). Results revealed that PALB2 gene expression in TAM-treated and control cell lines were respectively determined to be 7.1836 and 3.810.22.

## Discussion

This study revealed that TAM treatment can increase the expression of the PALB2 gene compared to the control group in the MCF7 cell line. Investigations indicated that PALB2 increases concurrently with the progression of breast cancer, which is rational due to its tumor suppression role [17][18][19]. Wu et al. (2020) found that PALB2 is the inseparable part of the required BRCA series for the process of DNA repair [19]. These findings may be explained by the fact that BRCA2 and PALB2 proteins are directly connected to BRCA1 and act as the DNA repair complexes [13]. Another interpretation could be that there is a putative mechanism as to the PALB2 expresputative mechanism as to the PALB2 expression level regulation in the epigenetic system with CpG island methylation as the most relevant one. As an example, there is a 92.5% promoter methylation and this hypermethylated state is accompanied by an increase in PALB2 expression level in plasma samples of breast cancer patients [14]. In major studies, 18 target genes such as BRCA1, BRCA2, and PALB2 were reported as biomarkers for cancer progression and development [20][21]. Several investigations have tried to show the effect of TAM on gene expression at the transcriptome level [22][23][24]. However, not all molecular mechanisms and functions of TAM have been fully described [25]. According to the result of this study and insights into the mechanisms of TAM, it can be claimed that an increase in PALB2 gene expression under TAM therapy is one of the possible mechanisms to avoid cancer development.

BRIP1 gene with BRCA1/2 contributes to regulating DNA repair and cell cycle. However, BRIP1 has been shown to have a dual function of an oncogene and a tumor suppressor. As an example, the BRIP1 candidate gene is classified as an intermediate risk factor based on the relative risk [26]. Furthermore, the BRIP1 gene is known as a biomarker for breast cancer diagnosis and treatment monitoring. However, our results indicated that TAM had no significant effect on BRIP1 gene expression between control and TAM-treated cell lines. A possible explanation might be the one-time point evaluation of the BRIP1 gene expression. BRIP1 gene may be an early or late response gene.

In addition, TAM therapy is a targeted therapy that blocks estrogen receptors. Thus, activation of the estrogen signaling pathways involves many gene networks and protein-protein interactions that contribute to the regulation of gene expression [23].

Therefore, the investigation of gene expressions at the transcriptome level by RNA-seq data is crucially essential to describe the BRIP1 gene expression under TAM therapy.

Limitation

Here, the expression of two candidate genes under TAM treatment in the MCF7 cell line has been investigated. However, TAM can affect the expression of many candidate genes in breast cancer.

## Conclusion

The quantitative assessment of the PALB2 gene as a potential marker could be considered a non-invasive method for breast cancer in the processes of prognostic evaluations, screening, and treatment monitoring. The relationship between the amount of mentioned genes expression and drug influence can be explored more extensively through more accurate investigations, such as evaluating the whole transcriptome and RNA-seq, to be applied as appropriate biomarkers and consequently, improve the non-invasive diagnosis and treatment monitoring.

## Acknowledgments

The research grant was provided by the Research Deputy of Shiraz University of Medical Sciences (number: 22863). The funding body of the study did not play any role in the design, collection, analysis, interpretation of data, and writing the manuscript.

## conflict of interest

The authors declare that they have no conflict of interest.
